# Supplementary figures and images for: Identification of proteins exhibiting in vitro RNA chaperone activity through gradient profiling in the Lyme disease spirochete, Borrelia burgdorferi
Source: RNA Biol. 2026 Jun 3;23(1):1–23. doi: 10.1080/15476286.2026.2684395 (PMC13285594; doi:10.1080/15476286.2026.2684395)

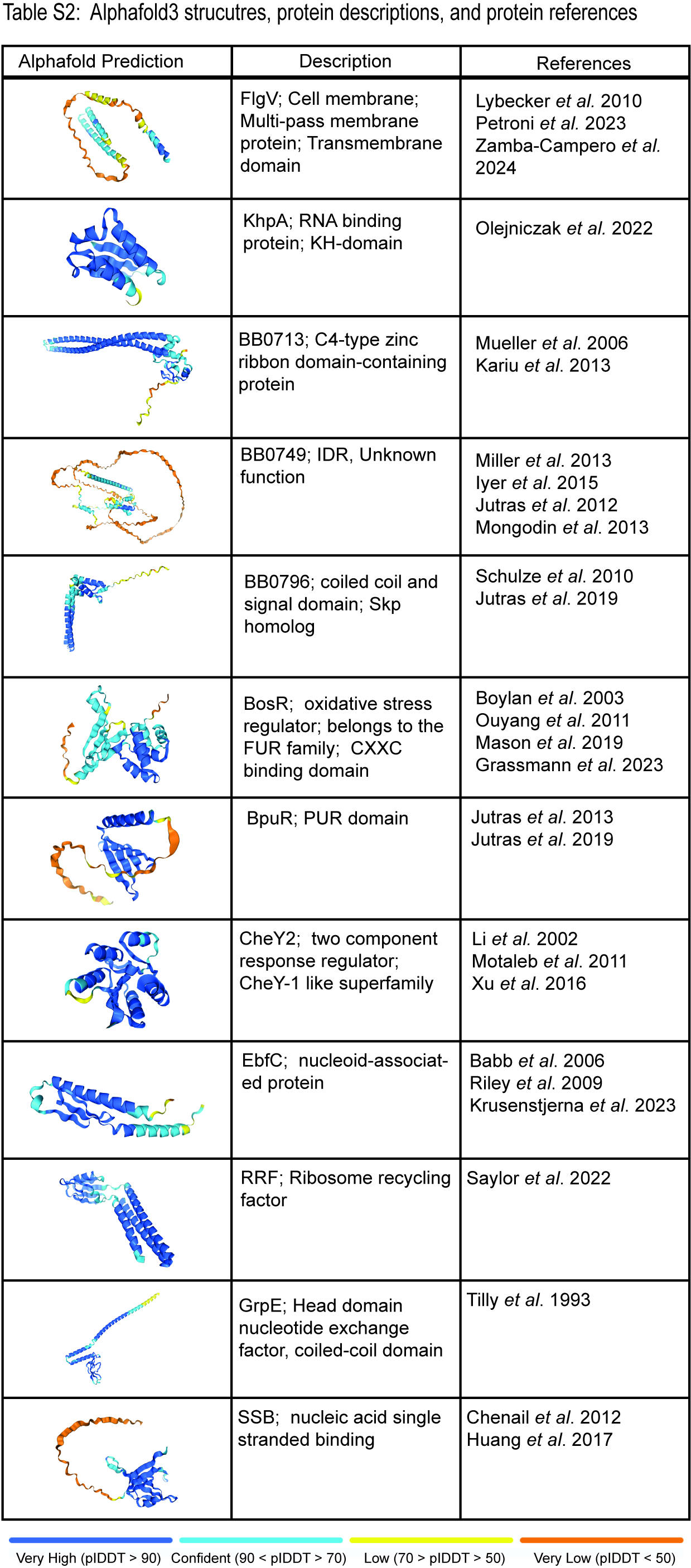

Supplement: Table S2.tif [file KRNB_A_2684395_SM7921.tif]

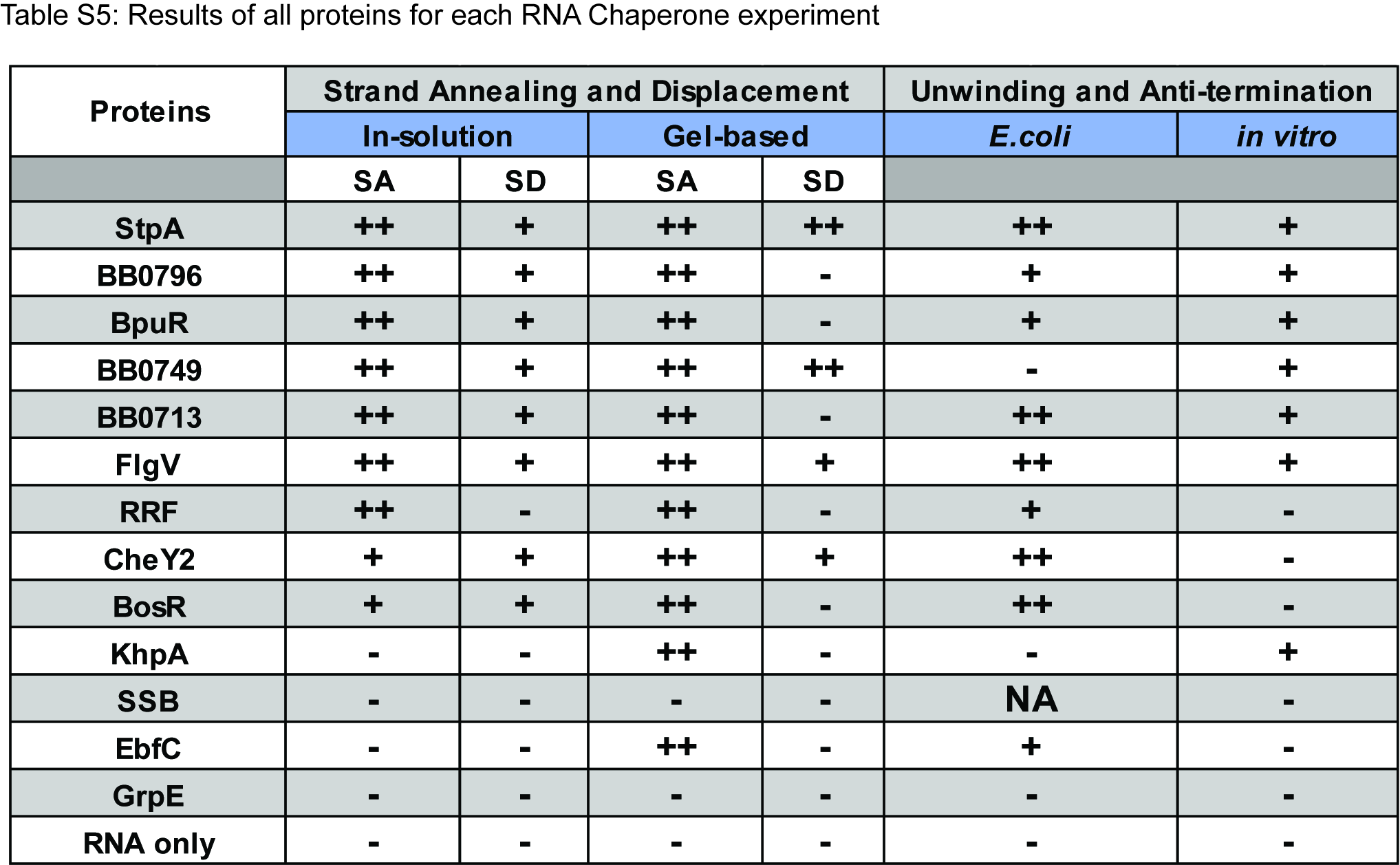

Supplement: Table S5.tif [file KRNB_A_2684395_SM7918.tif]
